# Sick Children

**Published:** 1877-09

**Authors:** 


### *SICK CHILDREN.*

There is no want of sympathy on the part of good people toward the sick and miserable in every condition of life; but we are painfully touched by the mute appeals of little sick children, expressed through their sad eyes and emaciated forms, that demand more than a careless sigh of regret for their sufferings. The thoughtful ask if the horrors of last summer are to be repeated now, and again next year, and forever? We are satisfied that the usual treatment employed by physicians in cases of "Cholera Infantum" is destructive of life, and that the ratio of deaths would be greatly diminished if the ordinary medication were abandoned and good nursing substituted, since it is impossible to convince the average doctor that opium destroys more children than all the diseases peculiar to infancy combined.

